# Impact assessment of barge trafficking on phytoplankton abundance and Chl *a* concentration, in River Ganga, India

**DOI:** 10.1371/journal.pone.0221451

**Published:** 2019-09-04

**Authors:** Soma Das Sarkar, Malay Naskar, Pranab Gogoi, Rohan Kumar Raman, Ranjan Kumar Manna, Srikanta Samanta, Bimal Prasanna Mohanty, Basanta Kumar Das

**Affiliations:** 1 Fishery Resource and Environment Management Division, ICAR-Central Inland Fisheries Research Institute, Barrackpore, Kolkata, West Bengal, India; 2 Kolkata Centre of ICAR-Central Inland Fisheries Research Institute, Salt Lake, Kolkata, West Bengal, India; 3 Riverine Ecology and Fisheries Division, ICAR-Central Inland Fisheries Research Institute, Barrackpore, Kolkata, West Bengal, India; 4 ICAR-Central Inland Fisheries Research Institute, Barrackpore, Kolkata, West Bengal, India; CNRS, FRANCE

## Abstract

Impact of barge movement on phytoplankton abundance and biomass was assessed in the lower stretch of river Ganga, popularly known as Bhagirathi-Hooghly river, during April 2016 to March, 2017. Based on the magnitude of tide, intensity of shipping and boating activities, the stretch from Baranagar to Lalbag (278 km), located at latitude (22°38'33.41"N to 24°10'59.75"N) and longitude (88°21'21.29"E to 88°16'5.65"E) was divided into three zones viz. zone—I (Baranagar to Barrackpore), zone II (Triveni to Balagarh) and zone III (Nabadweep to Lalbag). Water samples were collected randomly from six stations covering 22 barge movements at their passage at three different time intervals viz., 30 minutes before ‘barge movement’, during ‘barge movement’ and 30 minutes after ‘barge movement’. Analysis revealed the presence of 52 phytoplankton taxa belonged to 5 phylum during the study period. The abundance of phytoplankton was highest in zone—I followed by zone III and the zone II. A 44% decrease (1,997 ±1,510 ul^-1^) in phytoplankton abundance was observed during ‘barge movement’ with respect to normal condition (3,513 ± 2,239 ul^-1^) which could be due to propeller turbulence in the passage. Cell damage study revealed 21% damage in phytoplankton cell structure in ‘during barge’ followed by ‘after barge’ (10%) condition compared to natural state (6%). Study revealed that phytoplankton biomass (Chlorophyll *a*) was influenced by ‘barge movement’ in the sampling stretches and the impact was assessed by one way ANOVA. The effect was found significant at Barrackpore (p <0.01), Triveni (p <0.01), Balagarh (p <0.01) and Lalbag (p <0.01) where as it was insignificant at Baranagar and Nabadweep, which may be due to continuous and existing boat trafficking at Baranagar and Nabadweep. Two way ANOVA computed using ‘barge movement’ and sampling stations showed significant (p<0.01) effect on magnitude of Chl *a* concentrations in the sampling locations. Thus, the ‘barge movement’ influenced phytoplankton abundance and biomass, it had a detrimental effect on phytoplankton cell architecture also. The data set of this work serves as foundation information to understand the ecological implications augmented barge induced environmental disturbances in waterways. This is the first such study which depicts the impact of ‘barge movement’ on aquatic food chain linkages in Bhagirathi- Hooghly river.

## Introduction

The Ganga-Bhagirathi-Hooghly river system from Allahabad to Haldia port (1620 km) is known as the National Waterway No. 1 of India. It is the single most significant route of the country where cargo, passenger and cruise vessel are plied for the purpose of Inland Water Transport (IWT) [1].

For several years, the waterways have been exposed to various natural and anthropogenic pressures such as hydrological alterations [2], exotic invasion [3], metal and pesticide contaminations [4], commercial navigation, tourist and passenger boating activities, fishing operation etc. These stressors havelead to drastic change of the river ecosystem which in turn caused problem to the residents [5]. The intertidal zone of estuary also witnesses several changes due to vessel generated waves which resulted into changes in estuarine hydrodynamics including tidal regime [6]. Barge trafficking and navigation through water ways cause numerous effect on chemical and biological components of the river ecosystem along with hydrodynamic alterations [7], habitat destruction, changes in water quality [8], reduction of ichthyoplankton catch [9, 10], loss of production of larval [8, 11] and adult fishes [12].

Phytoplankton are aquatic primary producers and they are the bases of ecological pyramids. These floating communities are susceptible to the altered environmental condition [13]. Chlorophyll *a*(Chl *a*) is one among the photosynthetic pigments which play a prime role in photosynthesis [14, 15] and hence act as measure of phytoplankton biomass [16].

Episodic turbulences are high intensity turbulence in water, generated by the anthropogenic stresses such as rotational movement of boat propellers [17], and natural causes likestrong winds and breaking waves [18]. These are distinguished from ‘ambient’ turbulence based on magnitude of their intensity. Movement of barges generate turbulence in water which causes disturbance like alluviation and loose soil formation especially in shoreline of the rivers. Those loose soils are carried downstream by the river flow and also increase the suspended sediment loads. This unconsolidated soil is also accompanied with tree roots, clumps of grass from collapsed river banks. Consequently, eroded river bankcauses low light permeability in underwater, and prevents photosynthesis and phytoplankton development [19, 20] as well as micro-zooplanktonic growth [21]. Moreover, turbulence also triggers extracellular release of phytoplankton derived organic matter and trace metals upon turbulence exposure [17].

Navigation and barge trafficking have become an emerging concern for the ecosystem functioning and sustainability. Worldwide many studies have reported significant increase in algal productivity along with more than double numeric count of algal generic mean during termination period of barge trafficking in controlled river pools systems of Illinois river channel [8], reduction in invertebrate abundance[22], increasing bed of dead oyster in the intertidal zone [23]; mortality in gastropods and amphipods in central Europe [7]. Collision and destruction ofgreen algae in turbulent flow generated by oscillating grid apparatus reported in a laboratory experiment [24].The turbulence generated by shipping vessel (5x10^4^ cm^2^ s^-3^) is more than the natural turbulence generated by surface wave breaking (10^2^ cm^2^ s^-3^)[18, 25], and it causes mortality of phytoplankton (22%)[17] and copepod (30%) [26]. Gibson and Thomas [27] have opined that the intermittent episodic turbulence is responsible for decreased growth rate of phytoplankton as compared to those exposed to constant turbulence. Thus, the detrimental effect of turbulence has been reflected intoceasation of photosynthesis efficiency (20–30%)coupled with 32% reduction in diatom abundance and 22% increase in number of intact dead diatom cell in a simulated laboratory condition [17] and also had negative influence on dinoflagellates [28].There are few morereports on the alteration in the functional morphology ofphytoplankton assemblage in turbulent environment [29], benthic epifauna (insects and gastropods) in German lowland river [30] owing to barge induced environmental disturbances. In addition, effect of episodic turbulence on cell structure followed by mortality of diatoms [17], dinoflagellates [31] and copepods [26], larval perch [32] have been documented. Moreover, the harmful effects of excessive movement of recreational boat propeller on marine species diversity have also been well documented[33, 34]. The mentioned impact studies on phytoplankton are very specific to regulated flow condition such as river pools or turbulence generating devices in laboratory. Moreover,impact studies of barge traffickingin large river system are limited. Concurrently, making inferences on natural system by extrapolating the results obtained on the basis of simulated experiment may sometimes be inaccurate or may not mimic the natural systems. In a large river system, plankton abundance is subjected to spatial variation. It is worthwhile to take into account the spatial effect while investigating the influence of ‘barge movement’ on plankton abundance. Hence, the present study is attempted for the first time to quantify the phytoplankton diversity and cellular density and also to enumerate the proportion of broken phytoplankton cell due to ‘barge movement’ in a natural large river system, the Bhagirathi-Hooghly river of the mighty Ganga.

## Materials and methods

### Ethics statement

The present study on the estimation of phytoplankton abundance and biomass was performed in accordance with the approval by the Institute Animal Ethics Committee (IEAC) of ICAR-CIFRI vide approval no. CIFRI/ IEAC-16-17/03. No specific permission were required to collect phytoplankton samples at Bhagirathi-Hooghly river stretch (22°38'33.41"N, 88°21'21.29"E to 24°10'59.75"N, 88°16'5.65"E) in river Ganga.

### Study area

**Fig 1:**
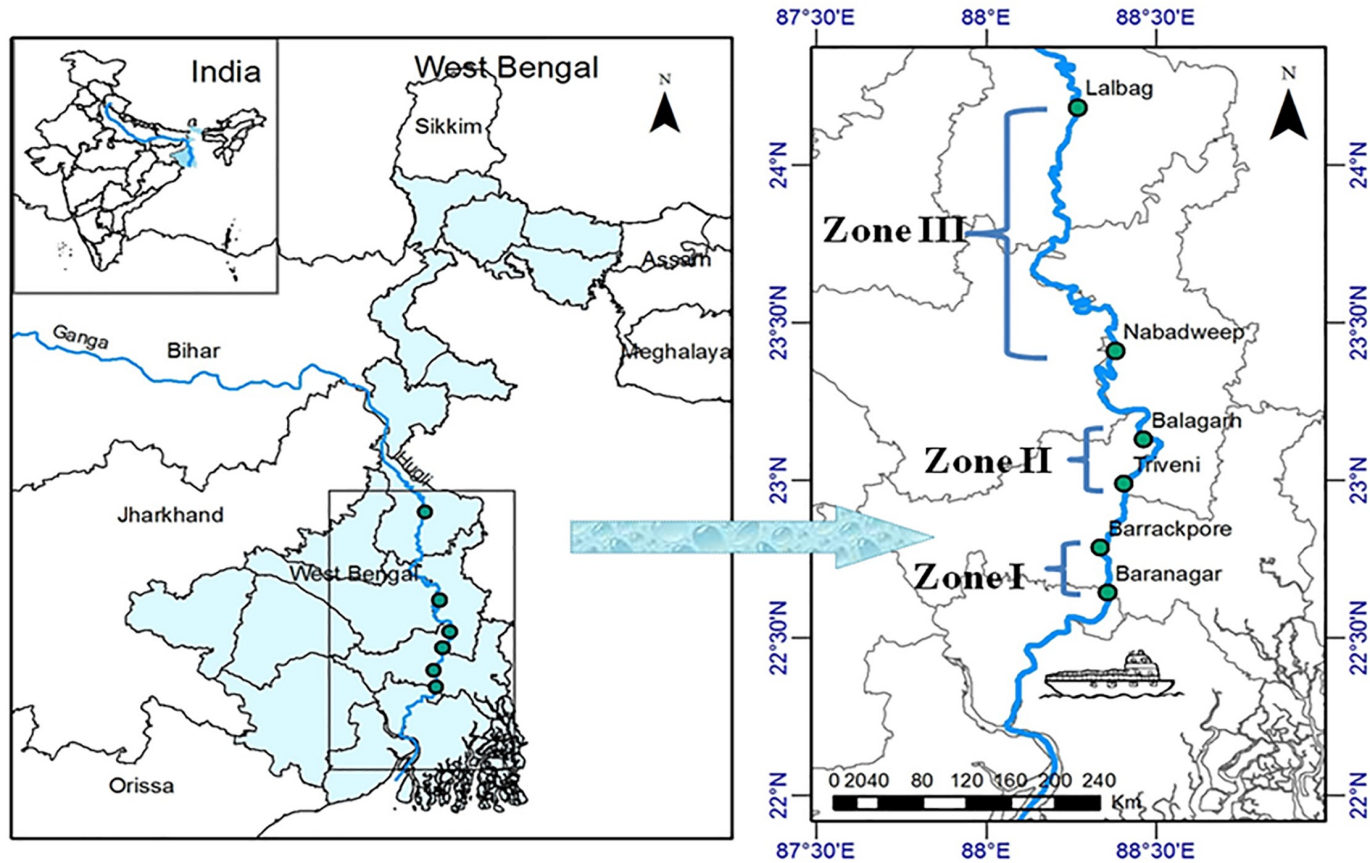
Sampling locations in Bhagirathi-Hooghly river stretch.

The shipping channel of the Bhagirathi-Hooghly river system was selected to study the impact of ‘barge movement’ on the phytoplankton dynamics in the lotic ecosystem. The study area is located in the freshwater zone and bounded between latitude 22°37'57.18" and 24°11'11.10" north and longitude 88°21'40.30" and 88°15'56.035" east. ArcGIS tool [35] was used to generate the study map that is portrayed in Fig 1. The 278 km span of studied river length stretches from Baranagar (22°38'33.41"N, 88°21'21.29"E) to Lalbag (24°10'59.75"N, 88°16'5.65"E).

## Sampling design

The study area was divided into three distinct zones selected by two steps strategies: (a) initial zones were subjectively chosen by inspecting visually the extent of disturbance activities, including commercial goods carrying vessels, tourist ships/ferry-boats, oil tankers, fishing activities, fishing boats etc.; (b) then final zonation was done objectively on the basis of a measurable quantity, the number of passenger ferry-boats per day. The description and measurable quantity, which was the basis of zonation, were as follows:

1. Zone I: Baranagar to Barrackpore with characteristics of intensive hydrodynamic disturbance activitiesand movement of 532 passenger ferry boats per day. This zone is influenced by high tidal effect.
2. Zone II: Triveni to Balagarhwith medium disturbance caused by fishing boats and movement of passenger vessels. The frequency of passenger boats was 262 per day. This zone witnesses moderate tidal effect.
3. Zone III: Nabadweep to Lalbag with least affected hydrodynamic disturbance activities caused mainly by movement of small passenger boats with frequency of 190 boats per day.This zone is designated as freshwater zone with no tidal effect.

Thereafter, two sites from each zone were selected to further accountwithin zone spatial variability. Thus a total of six sampling sites wereselected in the study area for ‘barge-movement’ dependent sample collection from each station.

## Algal sample collection and preparation

Water samples were collected in triplicate from subsurface (50 cm) water column from each of the six locations as mentioned in the sampling design, covering 22 barge movements along their passage, to study the microfloral diversity, abundance and biomass as well as physical damage of phytoplankton cell duringApril 2016 to March 2017. Water samples were collected by using standard water sampler based on design of ‘Ruttner water sampler’ [36]. The course of actions for collecting water samples at each site entails the following four sequential steps.

**Fig 2:**
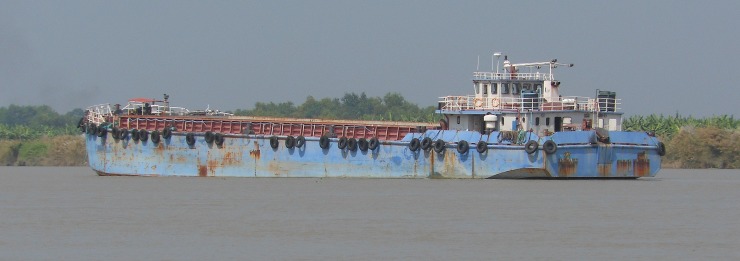
The barge in the National Waterway no. 1.

**Step 1**: Barges (72 m in length and 14 m in width) (Fig 2) were sailing/arrived in a specific time period in a day in the respective stations that we gathered information from Inland Water Authority of India (IWAI). The barges move with an average speed of 7–8 knots per hour at a frequency of 4 barges/day [1]. According to these prior informations, we marked the sampling spot on the shipping channel, and a motorized boat was anchored as close as possible at the identified spot.

**Step 2**: Water sample was collected before 30 minutes of arrival of the barge, and we named it as ‘before barge movement’ sample.

**Step 3**: The during ‘barge movement’ (during barge) samples were collected instantly when the moving barge cross parallel (barge come closest to the boat) to the sampling boat.

**Step 4:** After ‘barge movement’ (after barge) water samples were collected from the same spot after 30 minutes of departure of the barge.

For taxonomic analysis, samples were collected in 1 litre polyethylene bottles and fixed with lugol’s iodine solution (10 ml/l) and formaldehyde solution with a final concentration of 2% [20]. Algal samples were concentrated through sedimentation process by pouring the whole sample into a 1000 ml measuring cylinder and thereafter allowing the cylinder to stand on a vibration free surface for a period of 24–72 hours [37]. The sedimentation unit was maintained in a dark cool condition and kept away from heat. The top algal free water was drained out carefully. To minimize cell disturbance, the remaining top clear water was siphoned out using a 1000 μl pipette. The concentrated bio-volume was subjected to microscopy for analysis.

## Identification and enumeration of microalgae

Phytoplankton species were identified using Carl Zeiss Microscope with 400x, 630x magnifications and in 1000x magnification under oil immersion. Microphotographic data were recorded using a photo capture unit (Moticam 2300) digital microscopy. The phytoplankton were identified up to species level, wherever possible, by following [38–45]. Phyla were arranged following AlgaeBase website [46]. Quantum of abundance was measured using Neubauer-improved haemocytometer (Marienfeld, Germany) [47, 48].

### Evan’s blue (EB) staining

Evan’s Blue solution was used to count the broken cell under the light microscopy. Evan’s blue is a non-permeating dye generally used for cell viability assay. The dye enters cytoplasm and nucleus during membrane damage and colour the cell in blue. The stain was prepared by adding EB powder (HIMEDIA) with de-ionized water to make 2% w/v stock solution[49]. NaCl (0.5%) was used as preservative for the solution and 1X phosphate buffer saline (pH 7.4) was used to rinse the sample before cover slipping [50].

## Estimation of Chlorophyll *a* concentration

To explore impact of ‘barge movement’ on phytoplankton, water samples were collected in three replicates from the subsurface region (50 cm) and about 5 m (propeller depth) of the water column, following four step sampling strategy similar to algal sample collection.Samples were subjected to estimation of chlorophyll *a* biomass collected at three different time interval of ‘barge movement’ as mentioned. Chlorophyll *a* pigment was extracted with acetone and absorbance was measured at 664 nm, 647 nm and 630 nmusing a ultraviolet spectrophotometer (Thermo Spectronic UV1). Concentration of chlorophyll *a* (mg/m^3^ i.e. expressed from mg/l) was thus estimated [51].

## Statistical analysis

There were three primary sources of designed-based variation in the phytoplankton biomass as well as percentage of broken phytoplankton cell: (a) between and within zone spatial variation (b) variation over ‘sampling time’ according ‘barge movement’ and (c) variation over depth at each station, which was examined by applying separate one way ANOVA. The primary focus of the analysis was to evaluate the impact of ‘barge movement’ on the phytoplankton biomass and percentage of broken phytoplankton cell, while discarding the station or zone effect. Two way ANOVA was applied to accomplish this, in which six stations were designated as spatial treatments, accounting between and within zone spatial variability, and three ‘sampling time’ were considered as the treatments of ‘barge movement’. Since the treatment of ‘barge movement’ is dependent by design, we executed the analysis by incorporating the interaction effect between station and ‘barge movement’. Similar two way ANOVA was applied to test the significance of ‘barge movement’impact on percentage of broken phytoplankton cell; but,instead of stations, three zoneswere considered as the spatial treatments, while retaining the same treatmentsfor ‘barge movement’.Widely used LOESS smoothing technique, known as locally weighted scatterplot smoothing [52], was also used to generate predictive curve of phytoplankton biomass over the cumulative distance, considering BARANAGAR, the lowest downstream point, as origin. All the analyses were carried out in R software [53].

## Results and discussion

### Phytoplankton abundance and diversity

**Fig 3:**
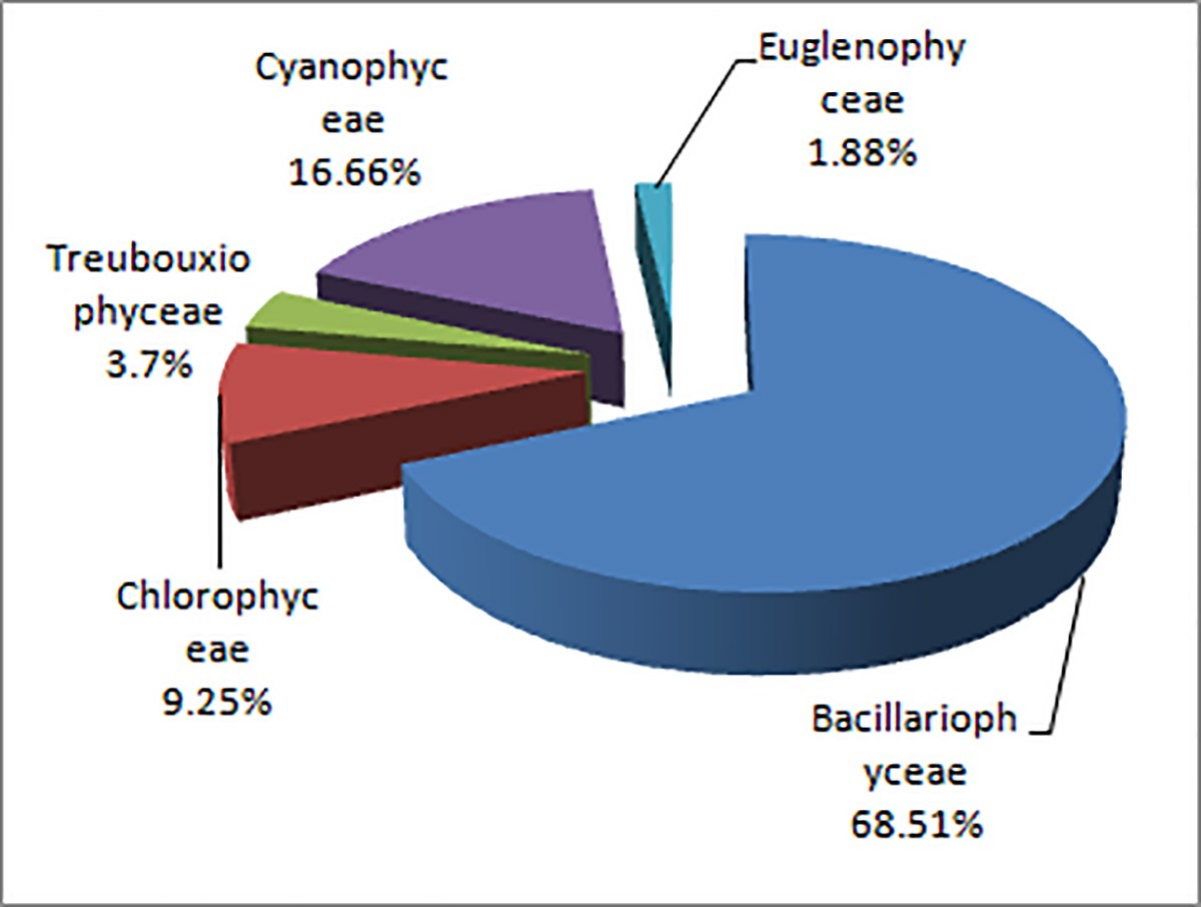
Compositions of algal group in the sampling stations.

**Fig 4:**
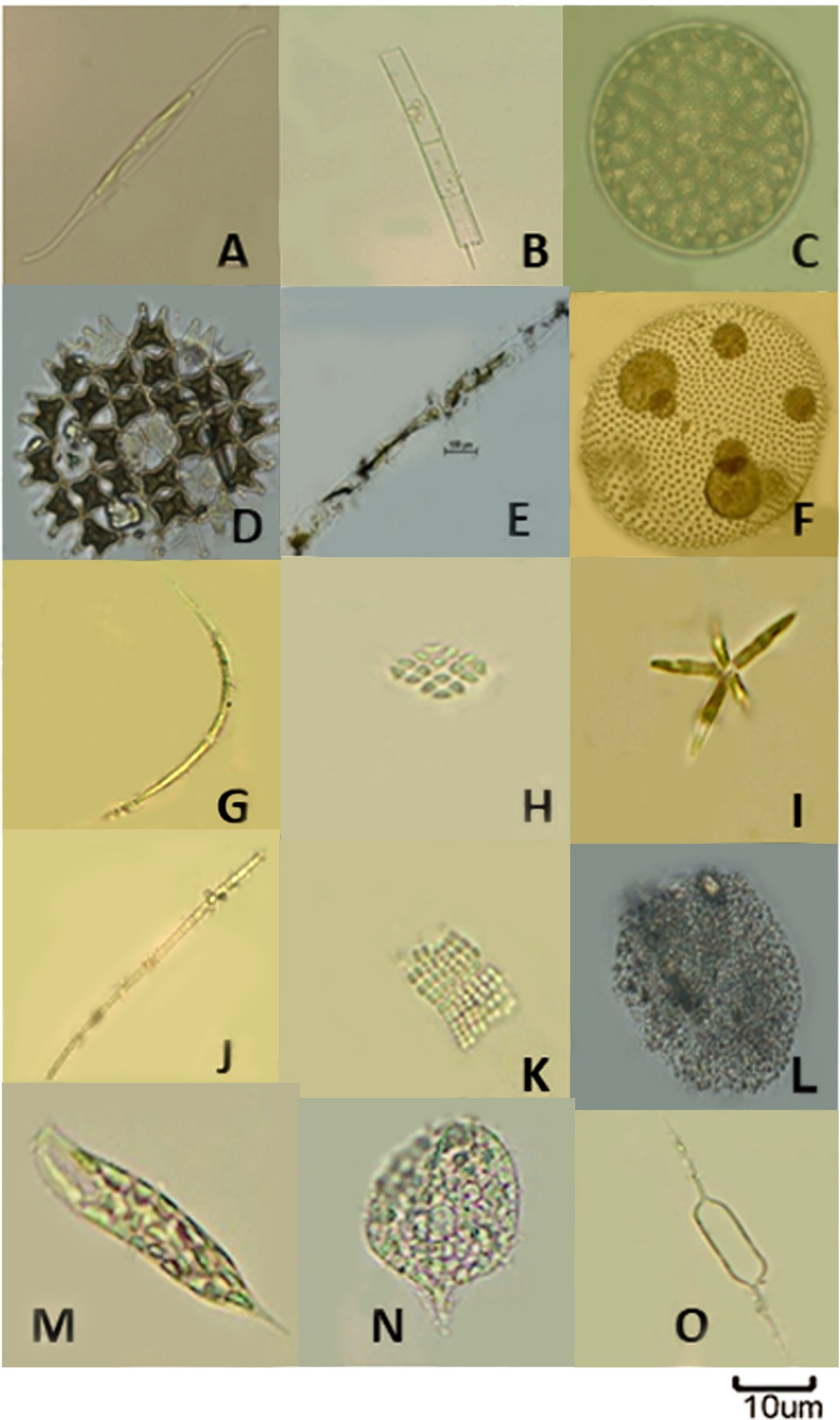
**Phytoplankton diversity—Bacillariophyta (A-C); Chlorophyta (D-I); Cyanobacteria (J-L); Euglenophyta (M-N); Ochrophyta (O).** Species in details: **A**. *Nitzschiareversa* **B.***Aulacoseiragranulata* **C.***Thalassiosira* sp. **D.***Pediastrum duplex* **E.***Mougeotia*sp. **F.***Volvox* sp. **G.***Closteriopsis* sp. **H**. *Crucigenia* sp. **I.***Actinastrum* sp. **J.***Anabaena* sp. **K.***Merismopedia* sp. **L**. *Microcystis* sp. **M.***Euglena* sp. **N.***Phacus* sp. **O.***Centritractus* sp.

A total of 52 taxa belonged to 5 phylum were recorded in Bhagirathi-Hooghly river stretch. The phytoplankton community was dominated by diatoms. Out of 52 identified taxa, twenty four were diatoms which comprised 18 pennales and 6 centrals. Pennate diatoms dominated in all the three zones during the study period. Compositions of five major algal groups were Bacillariophyceae (68.51%), Cyanophyceae (16.66%), Chlorophyceae (9.25%), Xanthophyceae (3.70%) and Euglenophyceae (1.88%) (Fig 3). Lowest species diversity was observed in class Euglenophyceae (two genera *Euglena* and *Phacus*). Most abundant centric diatoms were *Aulacoseira*, *Coscinodiscus*, *Cyclotella*, *Melosira* and *pennate* diatoms were *Navicula*, *Nitzschia*, *Cymbella*, *Synedra*, *Fragilaria* and *Gomphonema*. Chlorophyceae represented by 14 genera where *Spirogyra*, *Pediastrum*, *Ankistrodesmus and Coelastrum* were the most abundant genera. *Oscillatoria*, *Anabaena*, *Microcystis* and *Aphanocapsa* were the dominant cyanophytes in the sampling locations (Fig 4). Phytoplankton, experienced intermittent turbulence reflected lower growth rate as compared to those exposed to continuous turbulence [27]. The adverse impact of ‘barge movement’ on quantitative and qualitative abundance of phytoplankton was evident as observed cell abundance as well as diversity was lower after ‘barge movement’ than the before ‘barge movement’ situation. This is due to the effect of propeller turbulence in the barge passage. Analysis showed that a total of 38 taxa were recorded after ‘barge movement’ while 52 taxa before ‘barge movement’. Maximum density of phytoplankton was observed in the sample collected before ‘barge movement’ followed by after and during ‘barge movement’. The species composition varied in all three zones with predominance of *Aulacoseira*, *Cyclotella*, *Navicula*, *Nitzschia*, *Synedra*, *Spirogyra*, *Pediastrum*, *Ankistrodesmus*, *Anabaena*, *Oscillatoria* and *Microcystis* after ‘barge movement’. Abrupt changes in environmental variables influenced directly on phytoplankton community structure specially decreased tintinnid population during Annual Ganga Festival (AGF), Sagar Island, Sundarbans [54]. Present study showed that phytoplankton abundance was relatively more in zone I (lower stretch) followed by zone III (upper stretch) and the zone II (middle stretch) of the river. The phytoplankton taxa distribution is computed before and after ‘barge movement’ and the same has been represented in Table 1. Present study indicates higher abundance of diatoms in upper stretch (Zone III) followed by middle (zone II) and lower (zone I) stretch. Similar result was also reflected in previous study of ICAR- Central Inland Fisheries Research Institute [54, 55]. The diatom abundance observed in the present study differed from the earlier report of rare occurrence in the freshwater zone of Bhagirathi-Hooghly river [56]. Yellow green algae and Euglenophytes were less abundant in the present study. The quantitative abundance of phytoplankton ranged from 0.668 x 10^3^ ul^-1^ to 5.042 x 10^3^ ul^-1^ with highest abundance at Zone I before barge movement’, while lowest abundance was recorded at zone II during ‘barge movement’ (Table 2).

**Table 1:** Zone wise phytoplankton diversity.

Phytoplankton	Zone I		Zone II		Zone III	
**Phylum: Bacillariophyta**	Before barge	After barge	Before barge	After barge	Before barge	After barge
*Aulacoseiragranulata*	+	-	+	+	-	-
*Aulacoseira* sp.	++	+	++	++	++	+
*Cocconeis* sp.	-	-	-	+	-	-
*Coscinodiscus* sp.	+	-	++	-	+	-
*Cyclotella* sp.	+	+	+	+	+	+
*Cymbella* sp.	+	+	+	-	+	-
*Diatoma* sp.	-	+	-	-	-	+
*Diatomella* sp.	-	-	-	+	-	-
*Eunotia* sp.	+	-	-	-	+	-
*Fragilaria* sp.	+	-	+	+	++	-
*Gomphonema* sp.	+	+	-	+	-	-
*Gyrosigma*sp.	+	-	-	-	-	-
*Hantzschia* sp.	-	-	-	+	+	-
*Melosira* sp.	+	-	++	+	+	-
*Meridion* sp.	-	-	-	+	-	-
*Navicula* sp.	++	+	+	+	+	-
*Nitzschiaacicularis*	+	-	+	-	-	-
*Nitzschiareversa*	-	-	+	-	-	-
*Nitzschia* sp.	+	+	+	+	++	+
*Pinnularia* sp.	-	+	-	+	-	-
*Surirella* sp.	-	+	-	-	-	+
*Synedra* sp.	+	-	+	-	++	-
*Synedraulna*	+	+	+	-	+	+
*Thalassiosira* sp.	++	-	-	-	-	-
**Phylum: Chlorophyta**						
*Actinastrum* sp.	+	-	+	-	-	-
*Ankistrodesmus* sp.	+	-	-	+	+	-
*Closteriopsis* sp.	-	-	+	-	-	-
*Coelastrum* sp.	+	-	+	-	+	-
*Crucigenia* sp.	-	-	-	+	-	-
*Microspora* sp.	-	-	+	+	-	-
*Mougeotia* sp.	-	+	-	-	-	-
*Pediastrum duplex*	+	-	++	+	++	+
*Pediastrum simplex*	+	-	++	+	++	+
*Scenedesmus quadricauda*	+	-	-	-	+	-
*Scenedesmus* sp.	+	-	+	-	-	-
*Schizogonium* sp.	+	+	-	-	-	-
*Spirogyra* sp.	+	+	+	+	+	-
*Volvox* sp.	-	-	-	-	+	-
			
**Phylum: Cyanobacteria**						
*Anabaena* sp.	+	-	+	+	++	+
*Aphanocapsa* sp.	-	+	++	-	-	+
*Arthrospira* sp.	-	-	-	+	-	-
*Coelosphaeruim* sp.	-	-	+	+	-	-
*Merismopedia* sp.	+	-	+	-	+	-
*Microcystis* sp.	+	-	++	+	+	+
*Oscillatoria simplissima*	-	-	+	-	-	-
*Oscillatoria* sp.	+	+	+	+	++	+
*Phormidium* sp.	+	-	-	-	+	+
**Phylum: Ochrophyta**						
*Centritractus* sp.	-	-	-	+	-	-
*Gloeobotrys* sp.	-	-	+	-	-	-
*Tribonema* sp.	-	-	-	+	-	+
**Phylum: Euglenophyta**						
*Euglena* sp.	-	-	+	+	+	-
*Phacus* sp.	-	-	-	+	-	-

‘+’ indicates presence; ‘-’ indicates absence; ‘++’ indicates dominance

**Table 2:** Mean abundance of phytoplankton (mean ± SD) in different zones during different time interval.

Barge interval	Zone I	Zone II	Zone III	Average
**30 minutes before Barge movement**	5042 ± 734	668 ± 428	4831 ± 150	3513.66
**During Barge movement**	1146 ± 157	975 ± 893	3871 ± 141	1997.0
**30 minutes after barge movement**	2417 ± 165	710 ± 661	2769 ± 261	1965.33

Previous studies have reported an enormous fresh water discharge in Bhagirathi—Hooghly after commissioning of Farakka barrage in April, 1975 which changed the salinity regime [57, 58]. Gopalkrishnan [59] recorded 80 species of plankton in pre-barrage period which was decreased to 58 species during post-barrage period (1985–1991). The mean abundance of plankton recorded was 154.22 ul^-1^ during pre Farakka barrage (1959–62), but it was slightly higher (230 ul^-1^) during post Farakka barrage (1975–1991) in different zones of the Hooghly estuary [58]. Present study also observed low phytoplankton diversity (52 taxa) as compared to the pre-Farakka period, while relatively higher numerical abundance (3,513 ul^-1^) was recorded during normal condition (before ‘barge movement’) across the stations of Bhagirathi-Hooghly river. The phytoplankton abundance in Zone II depicted major decline (73%) with respect to Zone I. This decline might be the effect of effluents, a hindrance of phytoplankton production, released by Thermal power plant and Rayon industries located in Zone II. It was also observed that there were 77.27% and 19.86% declination in phytoplankton cell density during barge movement compared to normal condition within ZoneI and Zone III, respectively. The reduction of cell density in the selected sites of zone III might be due to least hydrodynamic disturbances.The *Coscinodiscus* sp., which was not recorded in earlier study at Nabadweep [60], was rarely present in Zone III predominated by freshwater. The mean quantitative abundance of phytoplankton in three different zones is summarized in Table 2.

The magnitude of water turbulence induced by boat propeller is more intense than the natural flow turbulence, and hence it causes mortality and physical stress of phytoplankton [17], resulting lesser abundance of phytoplankton during and after ‘barge movement’ than normal condition (before ‘barge movement’) in present study. The turbulence from small boats also have impact on the planktonic organisms. Bickel et al. [26] reported in excess of 30% mortality in natural copepod population when exposed to high intensity of boat trafficking. In the present study, there was 44% decrease in phytoplankton abundance during ‘barge movement’ with respect to before ‘barge movement’ situation.Since phytoplankton cells, especially the frustules of diatoms, have been broken/ damaged due to high speed rotational motion, the cells are distorted into tiny parts and lose their original frustules shape. As a result, minute parts of phytoplankton cells have not been identified, which resulted in decrease of numerical abundance. Moreover, due to turbulent motion plankton might have dispersed from the sampling sites (barge channel) or have drifted away due to increased thrust and tidal waves induced by the movement of the barge propeller.

## Impact of ‘barge movement’ on phytoplankton cell

**Fig 5:**
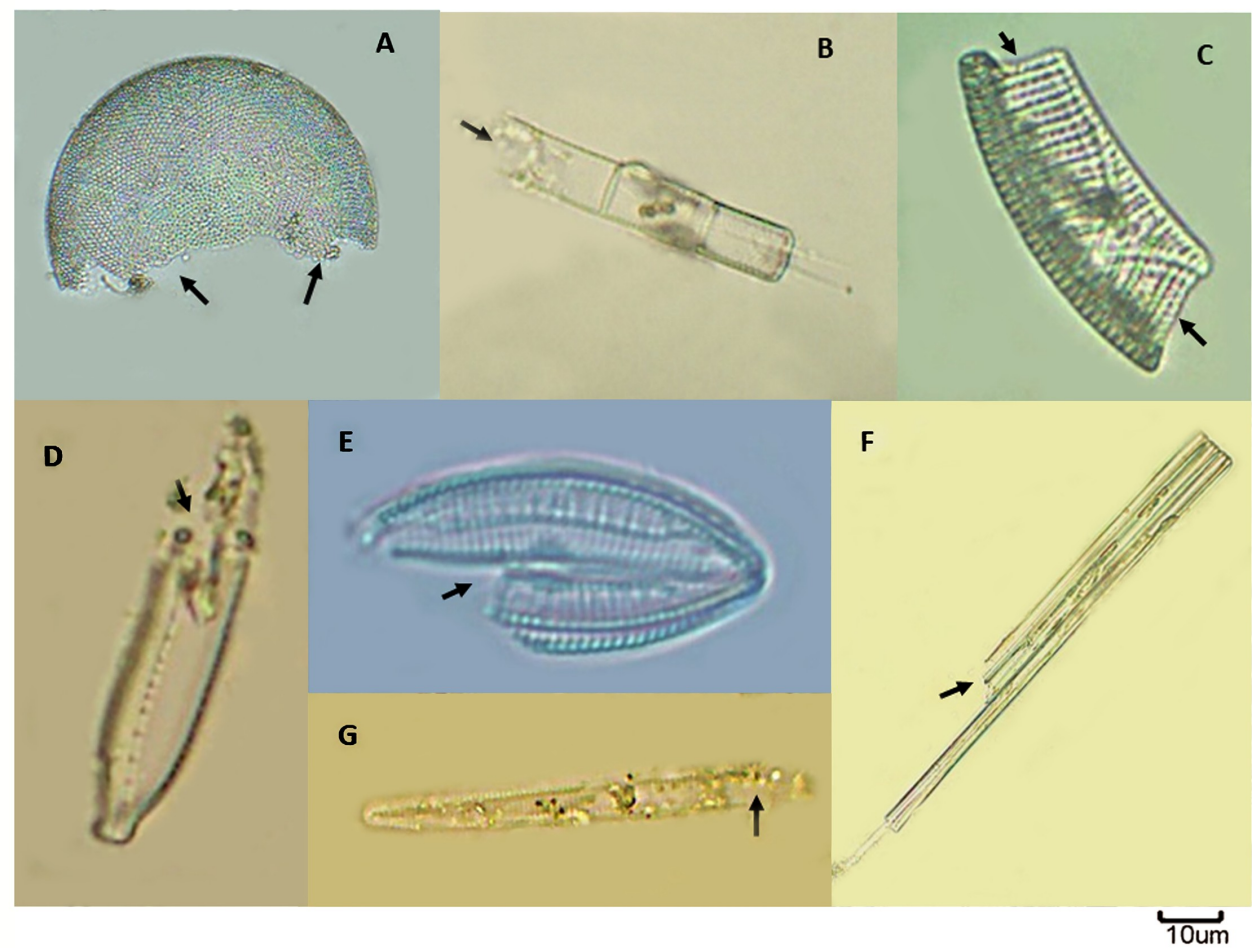
Broken phytoplankton cells. **A-***Coscinodiscus* sp.; **B-***Aulacoseira* sp.; **C-***Epithemia* sp.; **D-***Hantzschia* spp.; **E-***Mastogloia* sp.; **F-***Fragilaria* sp.; **G-***Synedra* sp. The broken parts are indicated by arrow heads.

During the study period, significant impact of ‘barge movement’ was recorded in phytoplankton cell structure in different locations. The damaged phytoplankton cells are represented in the form of percentage of broken or ruptured cell in the samples. The broken cell variation was found statistically significant at 5% level at different time and space (Table 3). The interactions between sampling interval and different zones were also found significant at 5% level. Study on damaged phytoplankton cell revealed, a total of 21.01% broken cell during barge and 10.22% damaged cell after ‘barge movement’ with respect to the natural condition (5.9%) in the entire study stretch (Table 4). Before ‘barge movement’, mean of ruptured cell percentage in middle zone (10.34) was significantly higher (p<0.05) than upper (4.45) and lower (2.87) zones, the difference was insignificant between lower and upper zones. During ‘barge movement’, the broken cell percentage in middle zone was significantly higher (26.25%; p<0.05) as compared to the lower (15.86) and upper (20.92) zones, while, no significant difference was observed between lower and upper zone. Similar results were also observed after the ‘barge movement’. The broken cell percentage in middle zone (14.11) was higher than upper (8.61) and lower (7.95) zone at 5% level of significance. The broken phytoplankton cells are shown in Fig 5.

**Table 3:** Impact of ‘barge movement’ on plankton cell fragmentation in the river.

Source of variation	df	Mean square	P value
**Barge movement**	2	151.7	<0.05
**Zone**	2	510.0	<0.05
**Interaction (Barge movement x Zone)**	4	89.9	<0.05
**Residuals**	18	36.9	

**Table 4:** Percentage of damaged cell (Mean ± SD) in three zones at different time interval of barge passage.

Barge movement	Lower zone	Middle zone	Upper zone	Average
**Before**	2.87±0.593	10.34±0.083	4.45±1.107	5.90
**During**	15.86±2.937	26.25±1.624	20.92±1.647	21.01
**30 minutes later**	7.95±0.054	14.11±1.684	8.61±0.290	10.22

**Fig 6:**
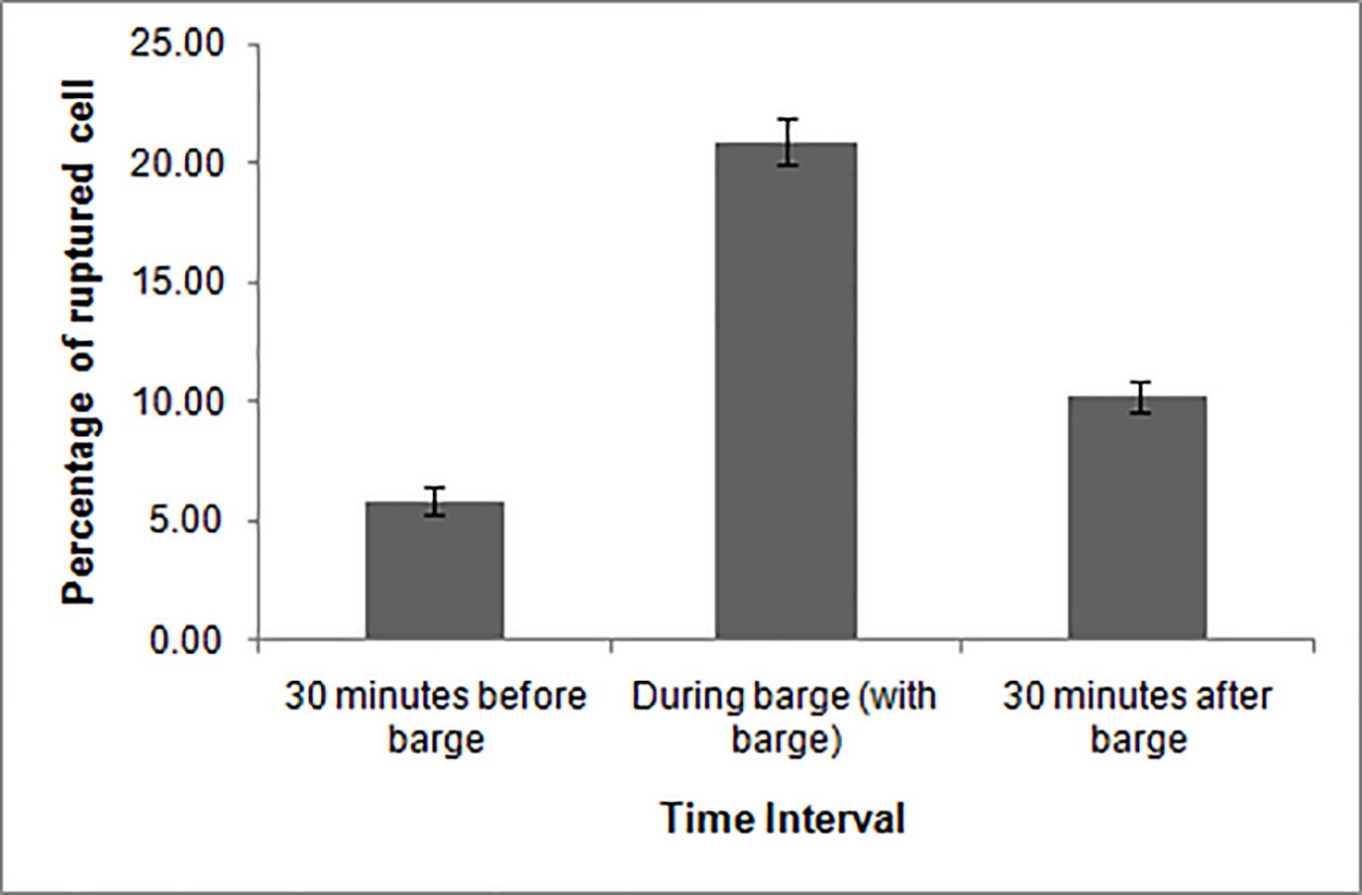
Quantum of phytoplankton cell with respect to barge damaged movement.

Study on the cell destruction and aggregation of dead cell of algae due to turbulent flow in laboratory experiment showed algal collision and cell destruction of *Scenedesmusquadricauda* with increase of level of turbulence in the water column [24]. Further, strong turbulence also act as causative agent for diatom mortality in terms of increase in number of dead cell [17]. Present study also reported 15% increase in percentage of ruptured cell of phytoplankton (Fig 6) during ‘barge movement’ due to higher turbulence in the water. The calculated percentage of damaged cell was reduced by 11% thirty minutes ‘after barge’ with respect to during ‘barge movement’ which might be due to continuous inflow of intact phytoplankton cellsin the lotic environment resulted into reduction of broken cell.The percentage of damaged cells was highest (26%) in the middle zone during ‘barge movement’. More concentration of damaged cell observed in this stretch can be attributed to the disruption of intracellular osmoregulatory mechanism of those marine/ brackish water phytoplankton species of that are being transported to this zone by tidal ingress [61] and abrupt creation of turbulence in the transporting passage. Present study showed 15% increase in broken cell during ‘barge movement’ which might be attributed to heavy turbulence generated by barge propeller. A negative effect of barge traffic on macrophytic vegetation such as *Chara* and *Potamogeton* spp. which has a direct effect on young recruits of coastal fish community has been reported in Stockholm Archipelago of Baltic Sea [62].

## Changes in phytoplankton biomass (Chl *a*)

One way ANOVA revealed that ‘barge movement’ influenced the Chl *a* biomass in the studied stretch both in time and space. Impact of ‘barge movement’ on phytoplankton biomass was found significant at Barrackpore (p <0.01) of lower zone, Triveni (p <0.01) and Balagarh (p <0.01) of middle zone and Lalbag (p <0.01) of upper zone (Table 5). The effect of depth on phytoplankton biomass was found non-significant (p > 0.09) among the sampling locations (Table 5). Therefore, data were pooled over depth for subsequent analysis.

**Table 5:** ANOVA table depicting impact of barge movement and depth on phytoplankton biomass (Chl *a*) at various stations.

Source of Variation	SS	df	MS	F	P-value
**Baranagar (BRNGR)**					
Depth	0.063029	1	0.063029	3.59664	0.094478
Barge movement	0.28704	8	0.03588	2.04744	0.165417
**Barrackpore (BKP)**					
Depth	0.028389	1	0.028389	0.94536	0.359378
Barge movement	3.901201	8	0.48765	16.23868	**0.000344**
**Triveni (TRVN)**					
Depth	0.001564	1	0.001564	0.01313	0.911565
Barge movement	6.135212	8	0.766901	6.44331	**0.008122**
**Balagarh (BLGRH)**					
Depth	0.465957	1	0.465957	3.95861	0.08182
Barge movement	4.64381	8	0.580476	4.93153	**0.018364**
**Nabadweep (NBDWP)**					
Depth	0.998244	1	0.998244	1.48367	0.257903
Barge movement	13.43654	8	1.679568	2.49631	0.108633
**Lalbag (LALBG)**					
Depth	0.034586	1	0.034586	1.30471	0.286386
Barge movement	2.125645	8	0.265706	10.0234	**0.001892**

**Fig 7:**
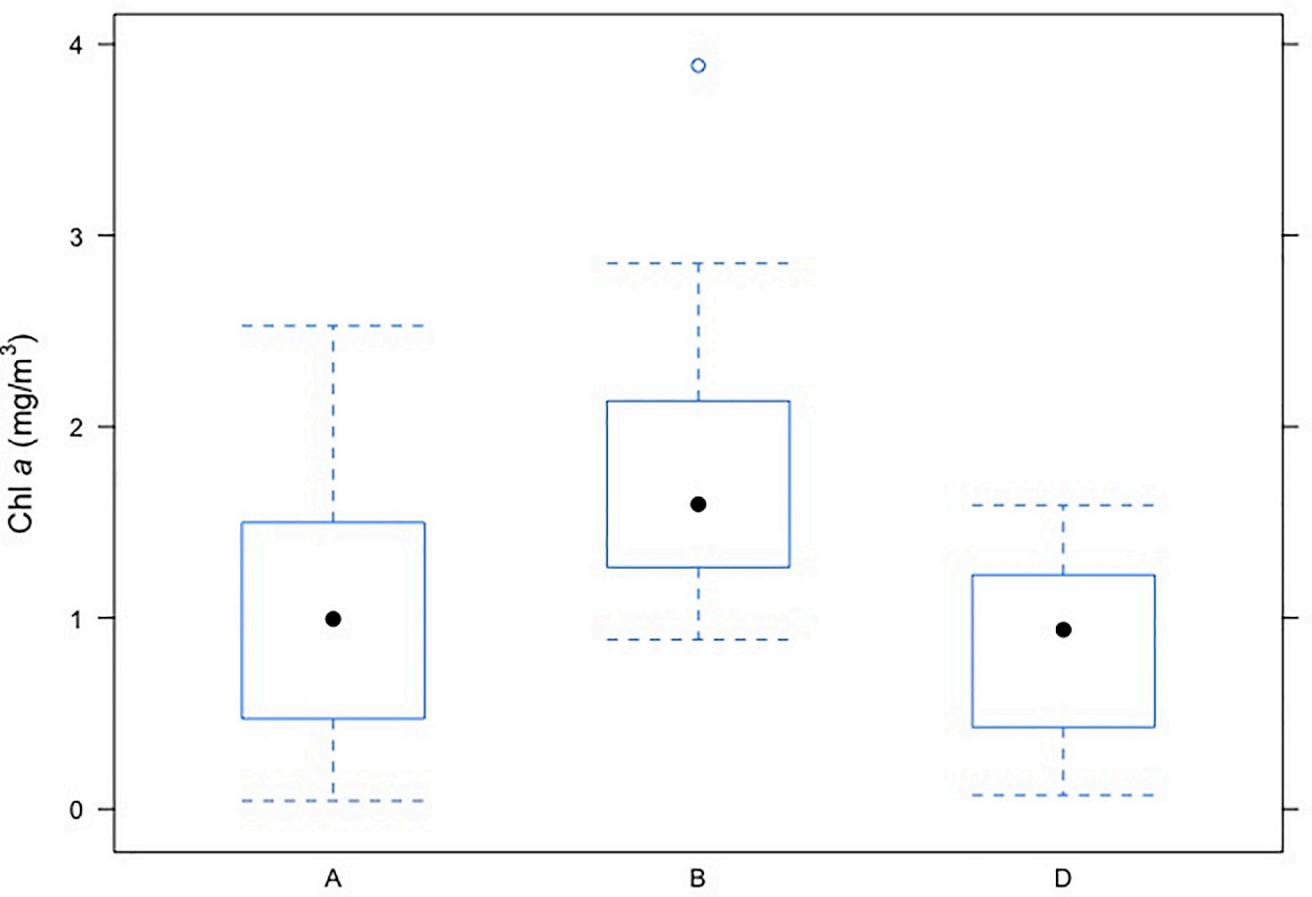
Box plot illustrating variation in phytoplankton biomass (Chl*a*) at various interval of barge movement. X axis depicts instances of Chl*a* data collection along Bhagirathi Hooghly river stretch with respect to ‘barge movement’; A: 30 minutes after barge; B: 30 minutes before barge, D: During ‘barge movement’; Y axis denotes Chl*a* biomass (mg/m^3^) at respective stations.

Further, impact of ‘barge movement’ and location on phytoplankton biomass was assessed by using two way ANOVA.Theresults showed (Table 6) that both the marginal effects of ‘barge movement’ and location were statistically significant (p<0.01). Therefore, concentration of phytoplankton biomass after averaging out over location was significantly different among ‘barge movement’ treatments: before (B) 30 minutes, during (I) and after 30 minutes (A) of ‘barge movements’(Fig 7). Similarly,locations had significant marginal impact on phytoplankton biomass, when averaged out over ‘barge movement’ effect.There was a considerable reduction of Chl *a* concentration during ‘barge movement’ (0.8756 mg/m^3^; 50.03%) as compared to before ‘barge movement’. This reduction was moderate (0.7326 mg/m^3^; 41.86%) after 30 minutes of ‘barge movement’. High turbulent motion of water generated by ‘barge movement’ facilitates to induce high turbidity and limited light penetration, which may be the two determinants, associated with reduced Chl*a* concentration [20]. Further, an impact study of turbulence on phytoplankton dynamics and Chl *a* concentration in Eastern English Channel showed that there was 3.5 fold decreases in Chl *a* in turbulence surf zone and inshore waters due to immediate wave breaking conditions followed by foam formation [63].Thus the significant reduction of chlorophyll concentration observed in the present study can be attributed to high water turbulence generated by ‘barge movement’, which is the cascading impact of ‘barge movement’ on the phytoplankton biomass.

**Table 6:** Two way ANOVA shown combined effects of barge movement and phytoplankton biomass.

Source of Variation	DF	Sum of Square	F statistics	P-value
**Barge movement**	2	15.886	47.160	<0.01
**Stations**	5	9.246	10.979	<0.01
**Barge movement x Stations**	10	8.947	5.3	<0.01
**Residuals**	90	15.158		

**Fig 8:**
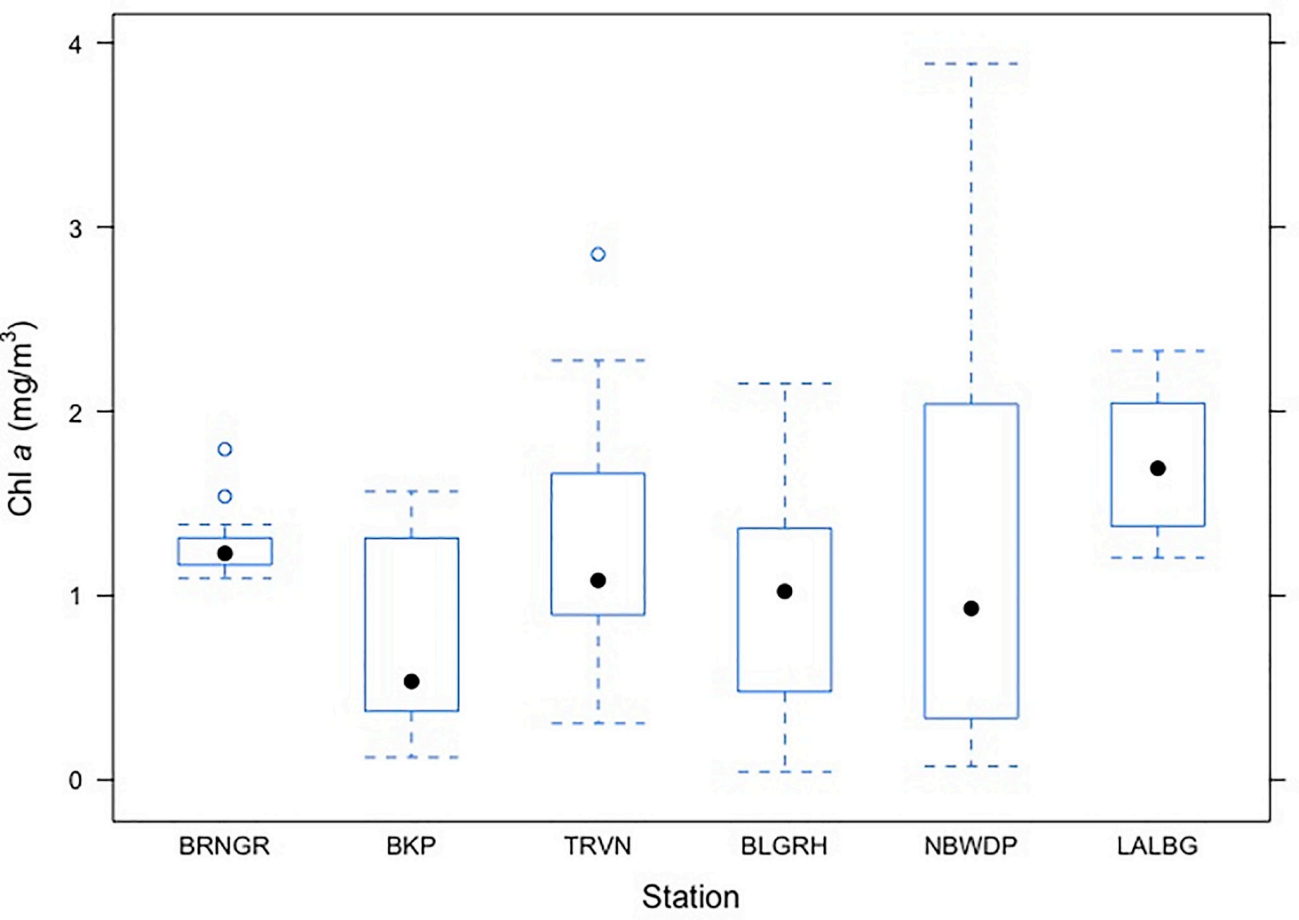
Box plot depicting Chlorophyll *a* concentration at different stations. X axis depicts sampling stations of Chl *a* data collection along Bhagirathi Hooghly river stretch; Sampling locations are abbreviated as follows—BRNGR: Baranagar; BKP: Barrackpore; TRVN: Triveni; BLGRH: Balagarh; NBDWP: Nabadweep; LALBG: Lalbag; Y axis denotes Chl *a* biomass (mg/m^3^) at respective stations.

**Fig 9:**
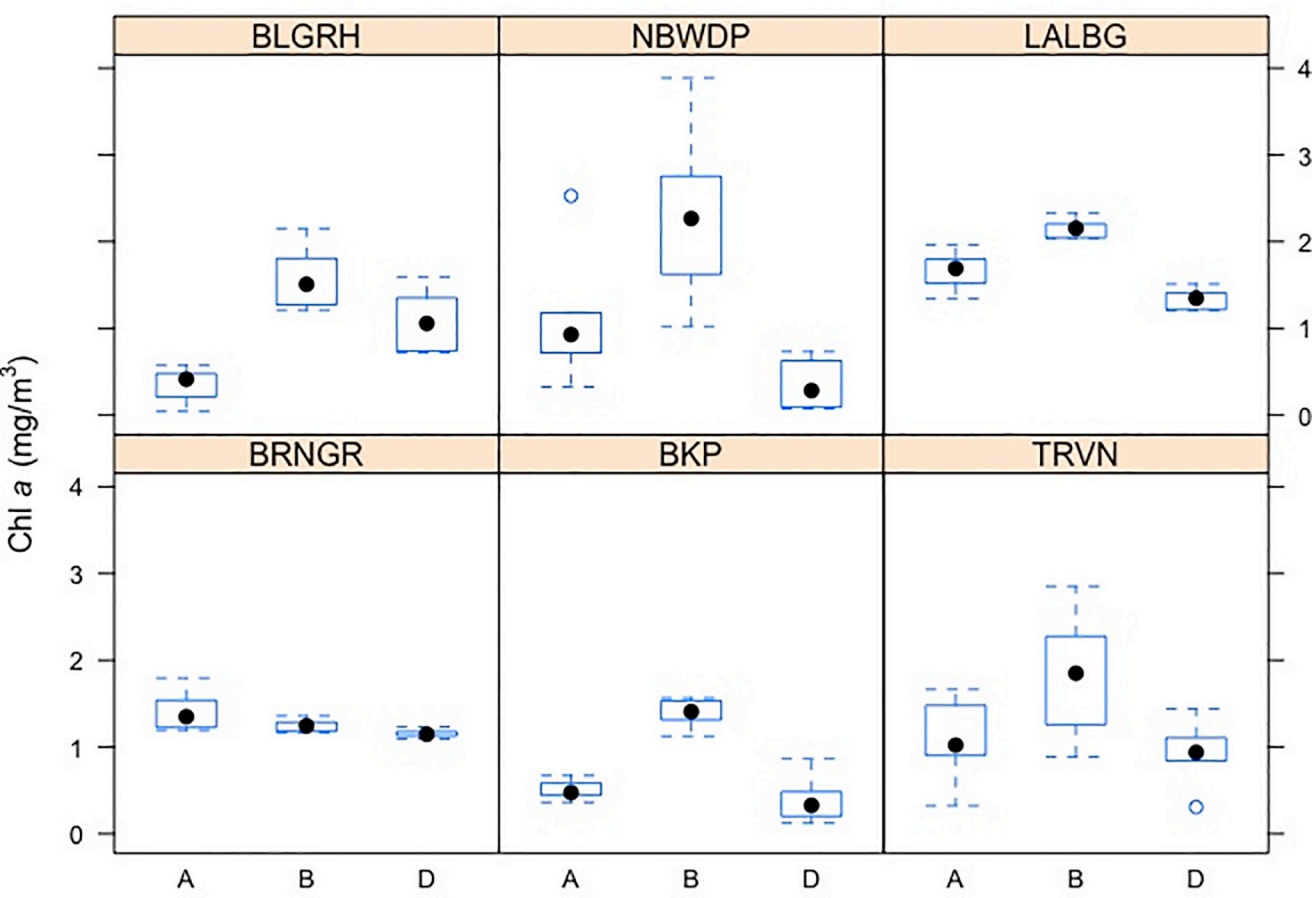
Effect of barge movement at each of the sampling station. Sampling locations are abbreviated as follows—BRNGR: Baranagar; BKP: Barrackpore; TRVN: Triveni; BLGRH: Balagarh; NBDWP: Nabadweep; LALBG: Lalbag. X axis depicts instances of Chl*a* data collection along Bhagirathi Hooghly river stretch with respect to ‘barge movement’; A: 30 minutes after barge; B: 30 minutes before barge, D: During ‘barge movement’;Y axis denotes Chl*a* biomass (mg/m^3^) at respective stations.

Marginal spatial effect on phytoplankton biomass was also evident, as concentration of phytoplankton biomass was relatively higher in Lalbag as compared to the rest of the sampling locations. The Chl *a* concentration at Triveni, Balagarh and Nabadweep was found to be similar during the study period while highest difference was observed between Lalbag and Barrackpore (Fig 8). The interaction effect between ‘barge movement’ and location also had an impact on phytoplankton biomass (p <0.01). There were significant variations of Chl *a* level among ‘barge movement’ treatment at eachlocations(Fig 9), excepting Baranagar, thereby indicating significant impact of ‘barge movement’ even within location.

**Fig 10:**
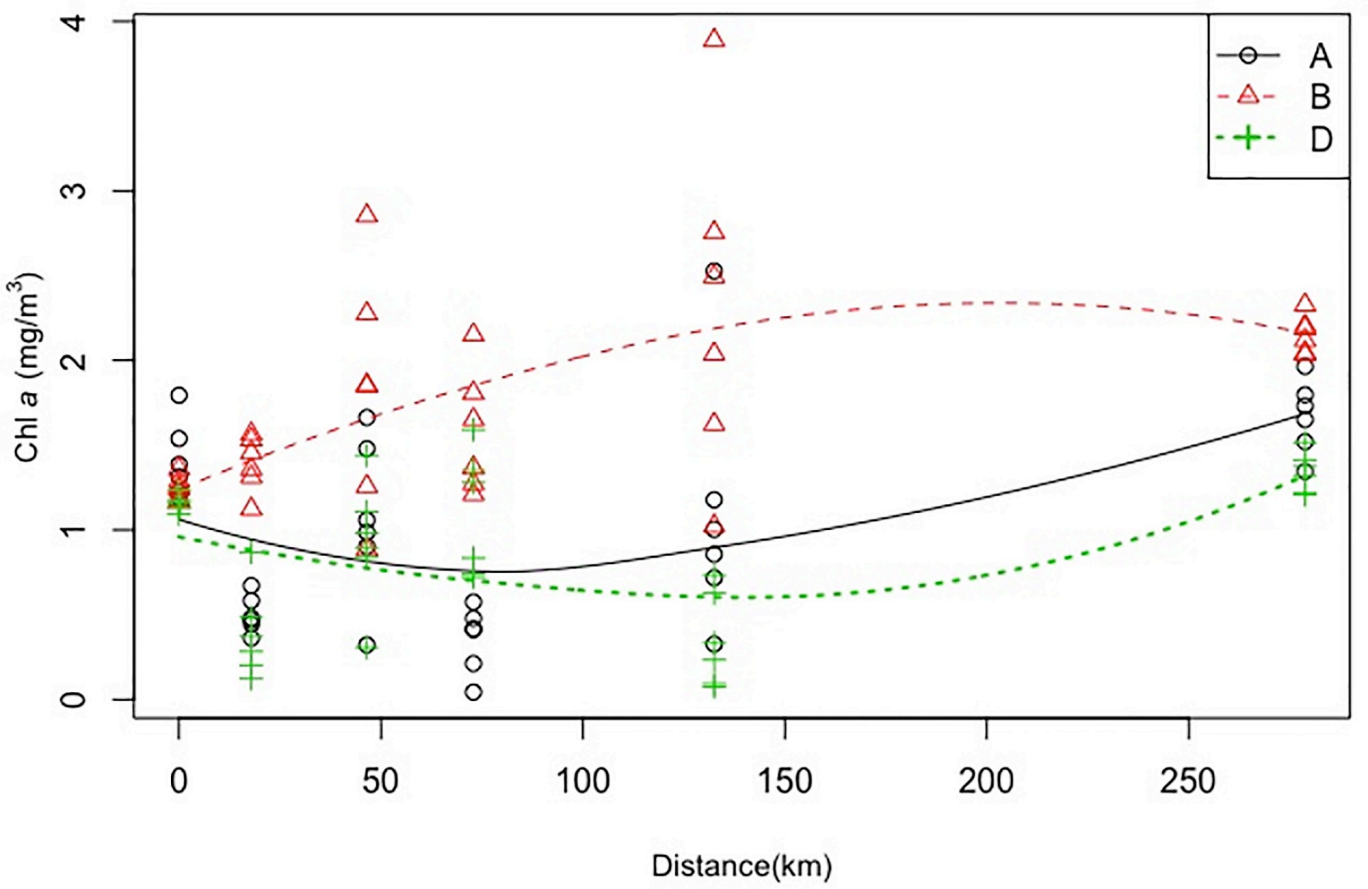
Scattered plot illustrates quantitative variation of Chl *a* biomass at various distance of sampling locations calculated from geographical coordinates. X axis depicts increasing distances of the sampling locations from station Baranagar as origin; Y axis denotes variation in Chl *a* biomass (mg/m^3^) at respective stations.

The cumulative distances of the sampling locations were calculated from Baranagar (22°38'33.41"N; 88°21'21.29"E) as origin using TerraMetrics Landsat Image in Google Earth. The illustration (Fig 10) depicts the predictive curve of phytoplankton biomass over cumulative distance. The impact on phytoplankton biomass was apparent due to movement of barges in Bhagirathi-Hooghly river. It is evident from the graphs that phytoplankton biomass before ‘barge movement’ (red colour) was higher throughout upstream distance as compared to during and after ‘barge movement’. The effect was relatively higher between 75 km to 200 km as compared to other regions due to low tidal effect and episodic disturbances in upper stretch coincides the higher phytoplankton biomass (2.273 mg/m^3^). It was found that difference of phytoplankton biomass between the sample collected during ‘barge movement’ and ‘after 30 minutes of ‘barge movement’ was low along the upstream distance.

In general, most phytoplankton species have a regeneration period of 2–4 days for increase of the population in a lentic ecosystem [64] and this doubling time become longer in a lotic water [65] which allows increase of quickly growing population with an exclusion of slow regenerating species due to horizontal flow movement. This condition may be intensified with frequent barge trafficking. Thus, thirty minutes interval after barge passage may not regain the normal condition of phytoplankton population. Impact studies on ichthyoplankton and small fishes due to vessel passage in Upper Mississippi river showed that reduction in mean catch of ichthyoplankton in surface and bottom water after 90 minutes of passage of loaded vessel downstream while short-term effects have been noticed in unloaded vessel immediately after passage in upstream [11]. Intensive barge movement along with several natural events such as strong wind and heavy waves also facilitate to increase the sediment load in the ecosystem. It causes low light permeability in the water column which limits photosynthesis and prevents phytoplankton development. This limiting factor damages the functionality of the primary producers which play a prime role in the aquatic food chain. Occurrence of high turbidity due to mass scale bathing of pilgrimage during AGF at Megadeltas of Sundarbans also resulted into sudden decrease of Chl *a* concentration (1.02 mg/m^3^) coupled with significant decrease in phytoplankton abundance (from 4.14 x 10^3^ cells l^-1^ to 2.997 x 10^3^ cells l^-1^) [54]. Worldwide several other studies have also highlighted the decreased phytoplankton growth rate owing to irregular and sporadic turbulence generated by boat propeller [19]. Since the negative growth of phytoplankton can also be attributed to anthropogenic pollution and natural stressors (strong winds and wave breakings) hence, intensive studies on impact of barge traffic on phytoplankton are advocated to understand the production biomass of primary producers.

## Conclusion

The present study has highlighted significant decrease in phytoplankton abundance and biomass in the natural river ecosystem due to the effect of movement of barges in the Bhagirathi-Hooghly river stretch. This is the first ever attempt to generate baseline information on impact of barge movement on photosynthetic autotrophs which are the primary producers of natural food web structure in the river. The similar study in a simulated laboratory condition with propeller turbulence intensities would give more accurate results in terms of estimating the detrimental effect of barge movement on the primary producers of an aquatic ecosystem. The pressure generated by the barge propeller and breaking of surface waves was not considered in the present study. Hence, a designed experiment in a simulated riverine condition with more intensive investigation is needed to understand the impact on photosynthetic efficiency and regeneration time of primary producers by episodic turbulence.

## Supporting information

S1 File Data_barge impact study on primary producers. (XLSX)
